# Structural studies of the PARP-1 BRCT domain

**DOI:** 10.1186/1472-6807-11-37

**Published:** 2011-10-03

**Authors:** Paul A Loeffler, Matthew J Cuneo, Geoffrey A Mueller, Eugene F DeRose, Scott A Gabel, Robert E London

**Affiliations:** 1 Department of Chemistry, Sam Houston State University, 1003 Bowers Blvd, Huntsville, Texas, 77340, USA; 2 The Laboratory of Structural Biology, National Institute of Environmental Health Sciences, 111 TW Alexander Dr, Research Triangle Park, North Carolina, 27709, USA

## Abstract

**Background:**

Poly(ADP-ribose) polymerase-1 (PARP-1) is one of the first proteins localized to foci of DNA damage. Upon activation by encountering nicked DNA, the PARP-1 mediated trans-poly(ADP-ribosyl)ation of DNA binding proteins occurs, facilitating access and accumulation of DNA repair factors. PARP-1 also auto-(ADP-ribosyl)ates its central BRCT-containing domain forming part of an interaction site for the DNA repair scaffolding protein X-ray cross complementing group 1 protein (XRCC1). The co-localization of XRCC1, as well as bound DNA repair factors, to sites of DNA damage is important for cell survival and genomic integrity.

**Results:**

Here we present the solution structure and biophysical characterization of the BRCT domain of rat PARP-1. The PARP-1 BRCT domain has the globular α/β fold characteristic of BRCT domains and has a thermal melting transition of 43.0°C. In contrast to a previous characterization of this domain, we demonstrate that it is monomeric in solution using both gel-filtration chromatography and small-angle X-ray scattering. Additionally, we report that the first BRCT domain of XRCC1 does not interact significantly with the PARP-1 BRCT domain in the absence of ADP-ribosylation. Moreover, none of the interactions with other longer PARP-1 constructs which previously had been demonstrated in a pull-down assay of mammalian cell extracts were detected.

**Conclusions:**

The PARP-1 BRCT domain has the conserved BRCT fold that is known to be an important protein:protein interaction module in DNA repair and cell signalling pathways. Data indicating no significant protein:protein interactions between PARP-1 and XRCC1 likely results from the absence of poly(ADP-ribose) in one or both binding partners, and further implicates a poly(ADP-ribose)-dependent mechanism for localization of XRCC1 to sites of DNA damage.

## Background

The DNA repair protein poly(ADP-ribose) polymerase-1 (PARP-1) is one of the first proteins localized to foci of DNA damage [1]. Although PARP-1 itself lacks any DNA-repair activity, interaction with damaged DNA stimulates the NAD^+^-dependent poly(ADP-ribosyl)ation activity of PARP-1 [2]. The trans-poly(ADP-ribosyl)ation of target proteins, including DNA packaging proteins [3], is postulated to reduce the DNA-binding affinity of these target proteins resulting in decondensation and accumulation of DNA-repair proteins to sites on the damaged DNA [4]. Poly(ADP-ribosyl)ation also regulates various proteins involved in cell cycle control [5], apoptosis [6] and transcriptional regulation [7]. PARP-1 is itself also a target of automodification by poly(ADP-ribose) which is important for the repair of alkylating agent-induced DNA damage [8]. The DNA damage detection, signalling, and recruitment roles of PARP-1 in maintenance of genomic integrity has made it an important target of anti-cancer therapies [9-11].

**Figure 1:**
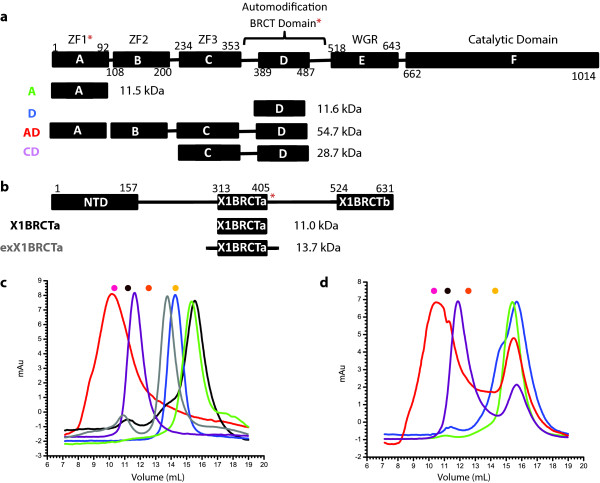
**Characterization of PARP-1 Constructs**. (a) Schematic of PARP-1 indicating the domain structure and constructs used in these experiments. Domain D corresponds to the PARP1 BRCT domain. Functional domains are indicated: ZF1, first zinc finger domain; ZF2, second zinc finger domain; ZF3, third zinc finger domain; WGR, WGR domain. Domains previously demonstrated to interact with XRCC1 are identified with a red asterisk. Molecular masses of constructs, including purification tags are indicated. (b) Schematic of XRCC1 domain structure and constructs used in these experiments. Domain previously identified to interact with PARP-1 is marked with a red asterisk. Molecular masses of constructs, including purification tags are indicated. (c) Gel-filtration chromatogram of domain constructs of XRCC1 and PARP-1. Plots are colored as in (a) (green, blue, red, or light purple) and (b) (black or gray). (d) Gel-filtration chromatogram of mixtures of X1BRCTa and the various PARP-1 domain constructs from panel (a). Chromatograms of the potential complexes are colored based on the scheme of the PARP-1 constructs identified in panel (a). Note that each of the curves in this panel exhibits two maxima that approximately correspond to the maximum positions in panel c and the maximum of the X1BRCTa in panel c (black curve). Molecular mass standards used are represented as colored circles (conalbumin (76.6 kDa), pink; ovalbumin (44.3 kDa), brown; carbonic anhydrase (29.0 kDa), orange; cytochrome C (14.3 kDa), gold) in panels (c) and (d).

Full-length PARP-1 is a six domain protein, with each of the six domains (A-F) encompassing a distinct functional role important in PARP-1 activation, localization and activity (Figure 1a). The first three N-terminal domains (domains A-C, residues 1-353), are zinc finger DNA binding domains with distinct functions in PARP-1 DNA-nick mediated activation [12-14]. The central, auto-modification region of PARP-1 (domain D), contains a BRCT domain (residues 389-487), as well as flanking segments containing glutamate [15] or lysine [16] residues that have been reported to serve as sites of auto-ADP ribosylation. Domain D is additionally implicated in mediating protein:protein interactions with the central BRCT domain of the DNA repair scaffolding protein X-ray cross complementing group 1 protein (XRCC1) [17,18], which in turn is constitutively bound to base-excision repair protein DNA ligase III-α [19] and interacts transiently with various DNA repair proteins [20-23] (Figure 1b). Adjacent to domain D, is a WGR domain (domain E, residues 518-643) followed by the catalytic domain (domain F, residues 662-1014), which possesses activities related to the ADP-ribose adduct formation, elongation, and branching activity, characteristic of this enzyme [24].

The auto-modification BRCT domain of PARP-1 is required for efficient repair of DNA damage; however, there has been limited biophysical characterization of this domain [18]. To better understand the PARP-1 BRCT domain and its role in recruitment of XRCC1-bound DNA repair factors, we determined the solution NMR structure of this isolated domain, characterized its solution behaviour using small-angle X-ray scattering and gel-filtration chromatography, and identified its thermal melting point with circular dichroism. As BRCT domains are known to form both homodimeric and heterodimeric protein interactions [19], and PARP-1 has previously been demonstrated to be a homodimer [18], we also investigated the quaternary interactions of the isolated PARP-1 BRCT domain. The PARP-1 BRCT domain is also implicated in mediating protein interactions with the central BRCT domain of XRCC1, and it has been unclear whether an interaction occurs in the absence of (ADP-ribosyl)ation of either binding partner [17,18]. In addition to the isolated PARP-1 BRCT domain, longer PARP-1 constructs comprising domains A through D, were prepared and studied to further elucidate this interaction between the central BRCT domain of XRCC1 and that of PARP-1.

## Results and Discussion

### Gel-filtration of the PARP-1 BRCT domain

BRCT domains are known to mediate both heterodimeric and homodimeric protein interactions and the PARP-1 BRCT domain was previously demonstrated to readily form a homodimer under physiologically-relevant conditions [18]. Gel-filtration chromatography of the PARP-1 BRCT domain (residues 389-487) was carried out to determine the quaternary state of the isolated domain. The PARP-1 BRCT domain elutes from the gel-filtration column at an apparent molecular mass of 12.5 kDa (Figure 1c), consistent with a monomeric quaternary state of the 11.6 kDa protein, and with our molecular mass determination using small-angle X-ray scattering data (below). The previous studies demonstrating that the PARP-1 BRCT exists as a homodimer [18] were carried out with a construct that had two purification tags (N-terminal 6-His/T7 Epitope). The construct was also longer at the N-terminus and was shorter by 16 amino acids at the C-terminus than the construct used in our experiments. It has been shown that the linker regions connecting tandem BRCT domains (Derbyshire et al., 2002; Joo et al., 2002), and more recently the linker region preceding an isolated BRCT domain (Cuneo et al., 2011), play important roles in complex formation. Therefore we carried out gel-filtration experiments with a construct consisting of domain C through the PARP-1 BRCT domain (PARP-1 C-D) in order to determine if the longer construct would promote formation of homodimers. The PARP-1 C-D construct elutes from the gel-filtration column at an apparent molecular mass of 32.0 kDa (Figure 1c), consistent with monomeric quaternary state of the 29.0 kDa construct. We postulate that the previously-reported homodimers of the PARP-1 BRCT domain may have been formed as a result of the purification tags used, or due to the exposure of hydrophobic surfaces resulting from truncation of the C-terminus.

### Protein interactions of the PARP-1 BRCT domain

The PARP-1 BRCT domain has been demonstrated to interact with the first BRCT domain from XRCC1 (X1BRCTa) [17,18]. We sought to characterize this interaction using gel-filtration chromatography. Addition of equimolar amounts of PARP-1 BRCT and X1BRCTa to the gel-filtration column produced a single peak with a small shoulder on the gel-filtration elution profile (Figure 1d, **blue line**). However, the apparent molecular mass derived from the peak positions was consistent with co-eluting monomers rather than with the formation of a stable dimer under the conditions used. Addition of equimolar amounts of PARP-1 C-D and X1BRCTa also produced two peaks in the gel-filtration experiments, with each peak position consistent with the corresponding monomeric component (Figure 1d, **purple line**).

Previous characterization of the interaction of X1BRCTa with PARP-1 BRCT indicated a simultaneous interaction of X1BRCTa with the A and D domains of PARP-1 [17]. Gel-filtration experiments were used to ascertain if a protein interaction could be detected with the PARP-1 A domain or the PARP-1 A-D domains (Figure 1d). Addition of equimolar amounts of PARP-1 A and X1BRCTa produced a single peak, consistent with co-elution of monomeric X1BRCTa and PARP-1 A. Addition of equimolar amounts of PARP-1 A-D and X1BRCTa produced two peaks, consistent with the peak position of each monomer (Figure 1d). Because the previous characterization of the interaction of X1BRCTa with PARP-1 employed an X1BRCTa construct that was longer at the N-terminus [17,18], we sought to determine if the inability to detect an interaction could be due to the absence of this specific region. In addition to extending the N-terminus of X1BRCTa by 12 amino acids, the C-terminus was also extended by 10 amino acids (exX1BRCTa) (Figure 1b). Interestingly, the longer construct exhibited a substantially greater level of expression, ~ 10-fold increase. However, as with X1BRCTa, no interaction could be detected with any of the PARP-1 constructs (data not shown). Although the predicted molecular mass for this longer XRCC1 construct is 13.7 kDa, it elutes from the gel-filtration column at an apparent molecular mass of 15.8 kDa (Figure 1c); X1BRCTa elutes from the gel-filtration column at an apparent molecular mass of 7.1 kDa compared with a predicted M.W. = 11.0 kD (Figure 1c).

### Thermal stability of the PARP-1 BRCT domain

**Figure 2:**
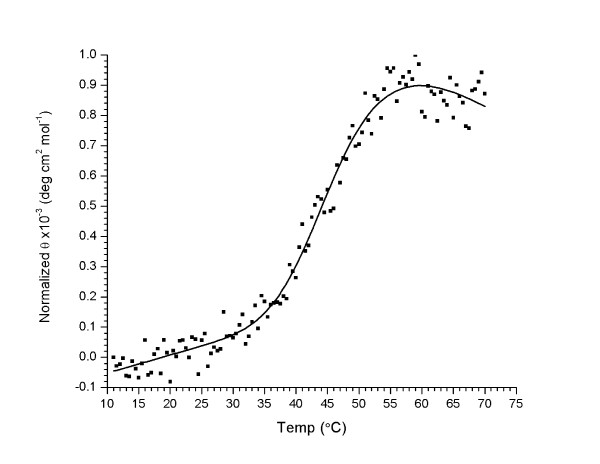
**Thermal melting of the PARP-1 BRCT domain**. Solid line is a fit of the circular dichroism signal to a two-state unfolding model.

Circular dichroism spectroscopy was used to assess the thermal stability of the PARP-1 BRCT domain. The ellipticity exhibited an irreversible thermal transition with a melting temperature of 43.0°C, suggesting that the domain is marginally stable at physiological temperatures (Figure 2). In full-length PARP-1, the linkers connecting our PARP-1 BRCT1 domain construct with the adjacent domains are relatively short; therefore it is possible that interactions with these domains are involved in stabilization of the BRCT domain when part of the full-length protein. Since the PARP1 structure is characterized by strongly electropositive regions on its surface (see below), and there are multiple sites of ADP ribosylation at residues adjacent to this domain [15,16], it is possible that this modification stabilizes the PARP1 BRCT domain.

### Small-angle X-ray scattering of the PARP-1 BRCT domain

**Figure 3:**
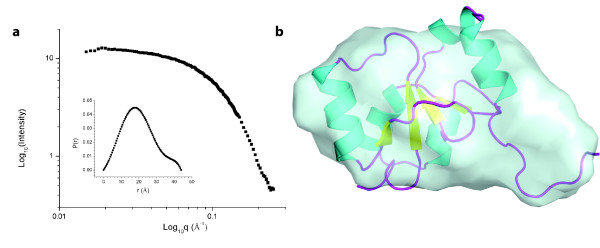
**Small-angle X-ray scattering of the PARP-1 BRCT domain**. (a) SAXS intensity data inset with the probability distribution function. (b) Ab initio molecular envelope (light blue surface representation) of the PARP-1 BRCT domain, generated from SAXS intensity data, superimposed with the NMR solution structure (ribbon representation).

The solution structure and quaternary state of the PARP-1 BRCT domain was investigated using small-angle X-ray scattering (Figure 3). Guinier analysis of the low q regions yielded an R_g _value of 15.4 ± 0.2 Å, averaged over three concentrations, with D_max _values of 44-46 Å. No significant variation in R_g _was observed upon dilution (Table 1). Comparison of the intensity at zero scattering angle (I_o_) of the PARP-1 BRCT, with a standard protein (hen egg lysozyme) was used to determine an apparent molecular mass of 10.3 ± 0.3 kDa over a concentration range 0.350 to 1.4 mM (Table 1). The experimentally determined molecular mass is similar to the mass calculated based on amino acid composition (11.6 kDa), indicating that this domain is in a monomeric state even at high concentrations. An *ab initio *model of the PARP-1 BRCT domain was constructed from the I(q) scattering data using the program GASBOR [25]. The resulting model has an overall globular shape consistent with the known fold of monomeric BRCT domains and not with models of dimeric BRCT domains (Figure 3b).

**Table 1:** SAXS data analysis

Sample	**I**_**o**_**/C**	MW (kDa)		**R**_**g(exp)**_	**R**_**g(calc)**_	**D**_**max**_
				
	Observed	Calculated	Expected	(Å)	(Å)	(Å)
**Lysozyme**	4.8 ± 0.1		14.3	15.1 ± 0.07	15.4	43
**PARP-1 BRCT (0.340 mM)**	3.6 ± 0.01	10.7	11.5	15.2 ± 0.03	14.8	44
**PARP-1 BRCT (0.685 mM)**	3.4 ± 0.05	10.1	11.5	15.6 ± 0.5	14.8	45
**PARP-1 BRCT (1.370 mM)**	3.4 ± 0.03	11.5	11.5	15.4 ± 0.1	14.8	46

### Solution structure of the PARP-1 BRCT domain

**Figure 4:**
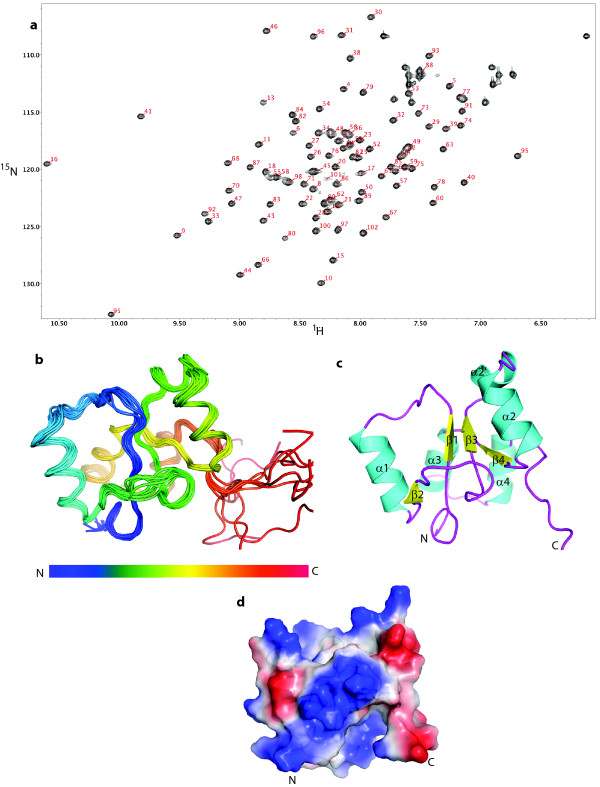
**NMR solution structure of the PARP-1 BRCT domain**. (a) ^1^H-^15^N HSQC spectra of the PARP-1 BRCT domain (assigned resonances indicated). Resonances corresponding to side-chain Asn and Gln residues are not labelled. (b) Ribbon diagram of the ten lowest energy structures in the NMR-generated ensemble of the PARP-1 BRCT domain. (c) Ribbon representation of the PARP-1 BRCT domain with labelled secondary structure elements. (d) Electrostatic surface representation of the PARP-1 BRCT domain (blue, positive; red, negative; white, neutral).

Structural characterization of BRCT domains provides the basis for understanding the roles of various residues in supporting intermolecular interactions [26,27]. We therefore sought to determine the three dimensional structure of the PARP-1 BRCT domain to gain insights into PARP-1 protein:protein interactions. Attempts to crystallize the PARP-1 BRCT domain were unsuccessful. The 600 MHz ^1^H-^15^N HSQC NMR spectrum is characterized by well dispersed resonances of even intensity indicating that in solution, the PARP-1 BRCT is folded and well behaved (Figure 4a). It apparently does not aggregate, and lacks significant intermediate conformational exchange behavior that would lead to resonance broadening. We thus were able to determine the solution structure of a U-[^13^C, ^15^N]-labelled 3.0 mM PARP-1 BRCT domain sample. All of the ^15^N and ^13^C assignments, and NOE data, were collected on a single sample. Not including the hexahistidine tag, the proton assignments were 93.5% complete as assessed by CYANA [28]. CYANA assigned 1775 NOE restraints as categorized in Table 2. The conformational ensemble was characterized by less than one NOE violation per structure. The restraints were converted to XPLOR-NIH format, and the structures were refined. Final statistics regarding the quality of the structures are provided in Table 2. The final XPLOR-NIH refinement [29] resulted in an ensemble of the ten lowest energy structures with a backbone RMSD of 0.5 Å and an all-heavy-atom RMSD of 0.8 Å for the ordered residues. This is compared with 2.6 Å and 3.1 Å RMSD for the backbone and heavy atoms, respectively, when considering the whole molecule. A total of 94.0% of structured residues are in the favored region of Ramachandran space and 5.0% are in the allowed Ramachandran region. A superposition of the ten lowest energy structures obtained from XPLOR-NIH is shown in Figure 4b. The solution structure determined by NMR and the calculated R_g _value of the NMR model (14.8 Å) is in agreement with the SAXS-based model (R_g _= 15.4 Å) (Figure 3b). At neutral pH, the PARP BRCT domain is strongly electropositive (calculated pI = 9.3), and the surface of the protein is characterized by several clusters of positively charged lysine residues (Figure 4d). This electrostatic surface, in combination with the reported polyADP-ribosylation of residues adjacent to the BRCT domain, suggests that there may be some type of electrostatic interaction between this surface and the polyADP-ribose backbone, perhaps acting to stabilize the structure in the absence of binding partners. Additionally, the fold of the rat PARP1 BRCT domain is essentially identical with that of the corresponding unpublished human BRCT domain (PDB code: 2COK), with which it shares 88.5% sequence identity.

**Table 2:** NMR Statistics

**NOE Restraints Assigned**^**1**^	
Intraresidue, |i-j| = 0	473
Sequential, |i-j| = 1	453
Short Range, 1 < |i-j| < 5	359
Long Range, |i-j|≥5	490
Total	1775
**Violations, Restraints**	
NOE	0.6 ± 0.5
Hydrogen Bond	5.4 ± 0.8

**Convergence (Å RMSD)**	
Structured Residues	
BB	0.5
Heavy Atoms	0.8
All Residues	
BB	2.6
Heavy Atoms	3.1

**Covalent geometry, RMSD**	
Bond (Å)	0.004 ± 0.0003
Angles (°)	0.62 ± 0.01
Impropers (°)	0.48 ± 0.01

**Ramachandran Space**	
Structured Residues	
Favored region	94%
Allowed region	5%
Outliers	1%
All Residues	
Favored region	87%
Allowed region	9%
Outliers	4%

**Clash Score**^**2**^	
Raw	22.7
Z-score	-2.4

1- Assigned by CYANA

2-From Molprobity

**Figure 5:**
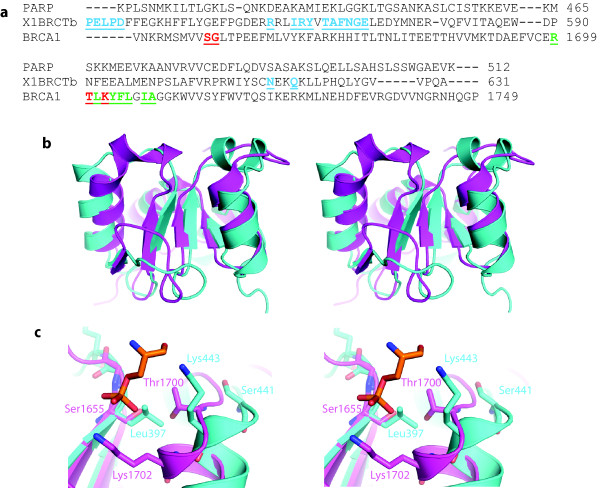
**Comparison of BRCT domains**. (a) CLUSTAL-W alignment of the PARP-1 BRCT domain (PARP), the second BRCT domain of XRCC1 (X1BRCTb), and the first BRCA1 BRCT domain (BRCA1). Residues contributing to the BRCA1 phospho-serine binding site are underlined and in red font; dual repeat interacting residues in the tandem BRCA1 BRCT domains are underlined and in green font, and XRCC1 homodimer interface residues are underlined and in cyan font (adapted from [31]). (b) Stereo view of a superposition of the structure of the sixth BRCT domain of TopBP1 (magenta) and PARP-1 BRCT (cyan). (c) Close-up stereo view of the superposition of the BRCA1 BRCT domain phosphoserine binding site (magenta) and the homologous region from the PARP-1 BRCT domain (cyan). Residues interacting with the phosphoserine (orange) in the BRCA1 BRCT domain (magenta), as well as structurally homologous residues from the PARP-1 BRCT domain (cyan) are shown in stick representation.

The PARP-1 BRCT domain has the globular α/β fold of a canonical BRCT domain, with a core of four parallel β-strands, surrounded by α-helices (Figure 4). The closest structural homolog based on a Dali analysis [30] is the sixth BRCT domain of TopBP1 (TopBP1 BRCT6) [31] (Figure 5b). PARP-1 BRCT and TopBP1 BRCT6 (pdb code: 3JVE) have a backbone RMSD of 2.3 Å and also share 22% and 64% amino acid identity and similarity, respectively. Interestingly, TopBP1 BRCT6 also interacts with PARP-1 and is itself a target of PARP-1 mediated ADP ribosylation [32], although no PAR binding to this domain could be detected in these previous studies. Similarly, in our studies no mono- or poly-ADP ribosylation of the isolated PARP-1 BRCT construct could be detected (data not shown), consistent with ADP-ribosylation occurring in the region adjacent to the C-terminus of the BRCT domain [16]. Although TopBP1 contains a degenerate phosphopeptide binding pocket, Leung et al. reported that the isolated TopBP1 BRCT domain does not appear to independently bind phosphopeptides [31]. A structural comparison of the binding pocket region of the first BRCA1 BRCT domain (pdb code: 1T2V[33]) with the corresponding region of the PARP1 BRCT domain indicates that the PARP1 domain would be even less likely to interact with phosphorylated peptides since the serine residue in the BRCA1 BRCT binding pocket is replaced by the Leu397 sidechain in our structure (Figure 5c). Additionally, the PARP-1 BRCT lacks residues consistent with either tandem or homodimeric BRCT interactions which characterize many members of this protein family (Figure 5a).

## Conclusions

The PARP-1 BRCT domain plays an important role in the localization of XRCC1 and in-turn, the XRCC1-complexed DNA repair factors, to sites of DNA damage. In order to better understand these functions, we have determined the solution structure of the rat PARP1 BRCT domain using both NMR and SAXS approaches. We also performed studies designed to elucidate its interactions with the central BRCT domain (X1BRCTa) of the scaffolding protein XRCC1.

It previously had been reported that under physiologically relevant conditions, the PARP-1 BRCT domain is a homodimer [18]. In contrast with expectations, both the gel-filtration and the SAXS studies reported here clearly indicate that this domain, even at high (millimolar) concentrations, is monomeric. We postulate that the previous identification of PARP-1 BRCT homodimers may have resulted from differences in the construct used in those experiments, which apparently terminated at residue Glu471, resulting in the loss of most of α-helix 4 and increased solvent exposure of many hydrophobic residues including Leu429, Val447, Val454, Val455, and Phe459. It is of interest that a homodimer was recently observed in the crystal structure of the PARP-1 domain C [14]. In this structure, the domain C monomers were in an anti-parallel orientation, suggesting that no quaternary contacts between two D domains can be made in the context of the full-length protein. Because the domain C homodimer was only observed in the crystalline state, rather than in solution, it is likely that additional segments of PARP-1 may be required to form a biologically-relevant PARP-1 homodimer. The PARP-1 BRCT domain also does not contain any of the specific structural motifs associated with either phosphopeptide, tandem, or homodimeric interactions, and likely will interact with its binding partners in a unique BRCT interaction mode.

The scaffolding protein XRCC1 is preferentially localized to sites of DNA damage when PARP-1 auto-ADP-ribosylation occurs [17]. The presence of XRCC1 results in ADP-ribosylation of XRCC1 and to a marked decrease in the catalytic activity of PARP-1, concomitant with the association of X1BRCTa and oligo-ADP-ribosylated PARP-1 [17]. It was unknown whether significant interaction between X1BRCT1a and PARP-1 occurs in the absence of ADP-ribosylated PARP-1. Our gel-filtration studies of PARP-1, which could not have been ADP-ribosylated because the constructs were bacterially expressed, do not detectably interact with X1BRCTa. This is in contrast to the observed association of these proteins when PARP-1, purified from mammalian cell culture, is oligo-ADP-ribosylated [17]. Based upon the current experiments and the studies of Masson et al. [17], the ADP-ribosylation of PARP-1 serves as a specific signal for association with XRCC1, and in-turn the recruitment of attached DNA repair proteins to sites of DNA damage.

In addition to the reported interactions of the PARP BRCT domain with XRCC1, studies demonstrating the interaction of the PARP BRCT domain with Hsp70 [34] and with nucleophosmin/B23 [35] have also been reported. Further studies are in progress to evaluate these possibilities.

## Methods

### Cloning, Over-expression, and Purification

A cDNA clone of the full-length rat PARP-1 gene was purchased from the ATCC. Several C-terminally, hexahistidine tagged constructs, consisting of the PARP-1 BRCT domain (residues 389-487), PARP-1 C-D (residues 234-512) and PARP-1 A (residues 1-92) were cloned into a pET21a vector. The PARP-1 A-D (residues 1-512) construct was an N-terminally tagged 6-His maltose binding protein fusion that was cloned using the Gateway system. The X1BRCTa (residues 313-405) and exX1BRCTa (residues 301-415) constructs were cloned into the GST-fusion vector pGEX4T3 that also contained a TEV protease site adjacent to the C-terminus of GST.

Plasmids were transformed into BL21-DE3-Rosetta cells (Novagen). Bacteria were grown in 2XYT broth overnight at 37°C. The overnight culture was diluted fifty-fold into fresh media and grown to an A_600 _of 0.8 at 37°C, and induced by the addition of 1 mM IPTG. The cells were grown overnight at 18°C, harvested by centrifugation (5000 g for 10 min), resuspended in an eluction buffer containing 20 mM imidazole, 500 mM sodium chloride, 20 mM Tris HCl (pH 7.5), and lysed by sonication. A clear lysate was prepared by centrifugation (30, 000 g for 30 min). Proteins were purified by immobilized metal ion affinity chromatography; 20 ml of lysate was loaded onto NTA-resin charged with Ni^+2 ^(Amersham). Following a wash with 20 ml of 20 mM imidazole buffer, the protein was eluted with a step gradient of 75 and 400 mM imidazole (500 mM sodium chloride, 20 mM Tris HCl, pH 7.5) buffers. Protein containing fractions were concentrated to 10 ml and loaded on a Superdex 26/60 S75 (Amersham) preparative grade gel filtration column that had been pre-equilibrated with a buffer consisting of 40 mM sodium phosphate (pH 7.5), 140 mM NaCl.

The X1BRCTa and exX1BRCTa fusion proteins were expressed as above. Lysed cells were re-suspended in a buffer containing 40 mM sodium phosphate (pH 7.5) and 140 mM NaCl. A clear lysate was prepared by centrifugation (30, 000 g for 30 min). Lysate was loaded onto a glutathione S-sepharose column that was subsequently washed with 30 mL of 40 mM sodium phosphate pH 7.5 and 140 mM NaCl. Protein was eluted with 15 mL of 10 mM reduced glutathione, 140 mM NaCl and 40 mM pH 7.5 Tris. The eluent samples were monitored for protein content at an absorbance of 280 nm. Samples were concentrated to 1 mL and digested overnight at 4°C with TEV protease. Cleaved GST and TEV protease were separated from the X1BRCTa or exX1BRCTa by gel-filtration chromatography.

### Gel Filtration and XRCC1 Binding Assays

All gel-filtration binding assays were carried out on an analytical grade Superdex 75 10/300 GL column that was calibrated with cytochrome c (14.3 kDa), ovalbumin (44.3 kDa), carbonic anhydrase (29.0 kDa) and conalbumin (76.6 kDa). A 100 μL aliquot of protein concentrate was loaded onto the column and was eluted with a buffer consisting of 40 mM sodium phosphate (pH 7.5), 140 mM NaCl at a flow rate of 0.8 mL/min. For the evaluation of protein:protein complex formation, proteins were mixed in an approximately a 1:1 stoichiometric ratio and incubated at room temperature prior to loading.

### NMR Assignments

The U-[^13^C, ^15^N]-labelled protein was expressed in minimal media containing ^15^NH_4_Cl and U-[^13^C] glucose and was purified as described above. Prior to NMR measurements, proteins were concentrated to ~3.0 mM and exchanged into 40 mM sodium phosphate pH 7.5, 140 mM NaCl, 5% D_2_O. The ^1^H-^15^N HSQC experiments were performed at 25°C on a Varian UNITY INOVA 600 MHz NMR spectrometer, equipped with a 5 mM ^1^H triple resonance cold probe with actively shielded z-axis gradients. The NMR data were processed using NMRPipe [36] and the spectra were analyzed using NMRView [37].

The sequential backbone and Cβ resonance assignments were made from analysis of HNCACB, CBCA(CO)NH, and HNCA experiments from the Varian BioPack pulse sequences. Side-chain proton and carbon chemical shift assignments were made from analysis of H(CCO)NH-TOCSY and (H)C(CO)NH-TOCSY spectra obtained using the Varian BioPack pulse sequences. Phenylalanine and tyrosine side-chain resonances were assigned from a combined analysis of (HB)CB(CGCD)HD, (HB)CB(CGCDCE)HE, and ^1^H-^13^C HSQC experiments from the Varian BioPack pulse sequences. All ^13^C and ^15^N NOE data were collected using the ^15^N-edited NOESY and ^13^C-edited NOESY, with a 100 ms mixing time.

### Structure Calculation

The NOE cross-peak information from the NOESY experiments, geometry restraints from TALOS [38], and hydrogen bond restrains based on prediction of secondary structure elements were input into CYANA [28]. Using its standard structure calculation protocol, CYANA assigned 1631 NOEs. The final assigned NOEs and the hydrogen bond information were reformatted for XPLOR-NIH, which was subsequently used for the final refinement [29]. Based on experience and the advice of G. Marius Clore (personal communication), the target distances and error bars for the NOE restraints were systematically increased prior to final refinement [26]. With XPLOR-NIH, one hundred structures were calculated and the ensemble of the ten lowest energy structures was deposited into the Protein Data Bank under accession code 2LE0. The NMR assignments were deposited in the Biological Magnetic Resonance Database under entry 17687.

### Small-angle X-ray scattering Data Analysis and Model Construction

The SAXS data were collected at beam line X9 at the National Synchrotron Light Source (Brookhaven National Laboratory). The wavelength of the beam was 0.953 Å. Fractions of PARP-1 BRCT were concentrated and dialyzed into a 10% glycerol, 15 mM NaPhosphate, 0.1 mM TCEP and 70 mM NaCl pH 7.5 buffer for SAXS analysis. Scattering data were circularly averaged and scaled to obtain a relative scattering intensity (*I*) as a function of momentum transfer vector, *q *(*q *= *4πsinθ*/*λ*), after subtraction of buffer scattering contributions.

All scattering data were analyzed using the Primus software package [39]; the GNOM45 software package [40] was used for all *P*(*r*) and *I_o _*analyses. Hen egg lysozyme was used as a standard reference protein for all I_o _analysis. Guinier plots were linear over a q-range of 0.012 to 0.088 Å^-1^. The three-dimensional shape of the PARP-1 BRCT domain was constructed from the SAXS data using the GASBOR22IQW program (*q*-range input for each analysis was from 0.01 to 0.24 Å^-1^) [25], by calculating the distribution of linearly connected 1.9 Å spheres that best fit the scattering data. Each calculation was repeated at least five times with different random starting points for the Monte Carlo optimization algorithm; no predefined shape or symmetry constraints were used. From these runs, the predicted structure with the lowest deviation of the calculated scattering profile from experimental data was used for interpretation. To compare the SAXS-based models with the atomic structures, the SUPCOMB13 [41] program was used.

## Authors' contributions

MJC and PAL carried out circular dichroism experiments. MJC, PAL and EFD carried out NMR experiments. MJC, PAL, GAM carried out NMR assignments and structure determination. MJC and SAG carried out SAXS experiments. MJC, PAL and REL designed and analyzed experiments and wrote the manuscript. All authors have read and approved the final manuscript.
